# Anxiety in synucleinopathies: neuronal circuitry, underlying pathomechanisms and current therapeutic strategies

**DOI:** 10.1038/s41531-023-00547-4

**Published:** 2023-06-22

**Authors:** Thuy Thi Lai, Birthe Gericke, Malte Feja, Michael Conoscenti, Moriel Zelikowsky, Franziska Richter

**Affiliations:** 1 Department of Pharmacology, Toxicology and Pharmacy, University of Veterinary Medicine, Hannover, Germany; 2 Center for Systems Neuroscience, Hannover, Germany; 3 University of Utah, Salt Lake City, UT USA

**Keywords:** Neurological manifestations, Parkinson's disease, Drug development

## Abstract

Synucleinopathies are neurodegenerative disorders characterized by alpha-synuclein (αSyn) accumulation in neurons or glial cells, including Parkinson’s disease (PD), dementia with Lewy bodies (DLB), and multiple system atrophy (MSA). αSyn-related pathology plays a critical role in the pathogenesis of synucleinopathies leading to the progressive loss of neuronal populations in specific brain regions and the development of motor and non-motor symptoms. Anxiety is among the most frequent non-motor symptoms in patients with PD, but it remains underrecognized and undertreated, which significantly reduces the quality of life for patients. Anxiety is defined as a neuropsychiatric complication with characteristics such as nervousness, loss of concentration, and sweating due to the anticipation of impending danger. In patients with PD, neuropathology in the amygdala, a central region in the anxiety and fear circuitry, may contribute to the high prevalence of anxiety. Studies in animal models reported αSyn pathology in the amygdala together with alteration of anxiety or fear learning response. Therefore, understanding the progression, extent, and specifics of pathology in the anxiety and fear circuitry in synucleinopathies will suggest novel approaches to the diagnosis and treatment of neuropsychiatric symptoms. Here, we provide an overview of studies that address neuropsychiatric symptoms in synucleinopathies. We offer insights into anxiety and fear circuitry in animal models and the current implications for therapeutic intervention. In summary, it is apparent that anxiety is not a bystander symptom in these disorders but reflects early pathogenic mechanisms in the cortico-limbic system which may even contribute as a driver to disease progression.

## Physiological and anatomical basis of anxiety and fear circuitry

Anxiety is a common human emotional experience with the potential to negatively impact the quality of life. In patients with synucleinopathies, the underlying pathophysiology of anxiety disorders remains poorly understood. There is, however, significant fundamental knowledge of brain physiology and anatomy responsible for fear and anxiety formation. The following sections will give an overview. For further details, the reader is being referred to the numerous recent reviews on the topics such as refs. ^1–4^.

### Concept of anxiety and fear

Anxiety is a generalized response to an unknown threat, while fear is focused on known external danger^5^. In physiological conditions, the main function of both fear and anxiety is to predict, react, and adjust to the signal of danger and threat^4^. Both are characterized by increased arousal, expectancy, autonomic and neuroendocrine activation, and specific behavior patterns. While fear and anxiety feel similar, they are distinguishable phenomena. From an evolutionary viewpoint, the term fear is used to describe feelings that occur when the source of the threat is either immediate or imminent, whereas anxiety is used to describe a feeling that occurs when the source of harm is uncertain or is distal in space or time^6^. According to that, anxiety disorders are a group of psychiatric disorders and a syndrome of ongoing anxiety^7^. Anxiety disorders comprise separation anxiety, selective mutism, specific phobia, social anxiety disorder, and generalized anxiety disorder^7^. The global prevalence of anxiety disorder in the general population is highly variable and ranged between 2.4% and 29.8% in the period 1980–2009^8^. According to the World Health Organization (WHO), in 2019, 301 million people were living with an anxiety disorder including 58 million children and adolescents. As with depression, anxiety disorders are more common among females than males (4.6% compared to 2.6% at the global level in 2015 according to the WHO).

Fear is commonly considered the biological basis of the emotion of all humans and many animals^9^. Pavlovian fear conditioning is commonly used to learn about the behavioral characteristics and neural mechanisms of fear acquisition^10^. This is a form of associative memory formation where a conditioned stimulus (CS) such as an auditory tone is paired with an aversive unconditioned stimulus (US) such as a foot shock. As a result, a memory is formed which allows the CS to elicit freezing, a behavioral index of fear. Fear extinction is an adaptive process whereby defensive responses are attenuated following the repeated experience of prior fear-related stimuli without harm^11^. Thus, interactions between fear conditioning and fear extinction shape behavior especially early in life^10^. In anxiety disorders, patients usually process fear-inducing information in excessive detail^12^. For instance, generalized anxiety disorders are characterized by an emotional state of excess and unrealistic worry, while panic attacks are characterized by repeated fear episodes.

### Anatomical basis of anxiety and fear

**Fig. 1:**
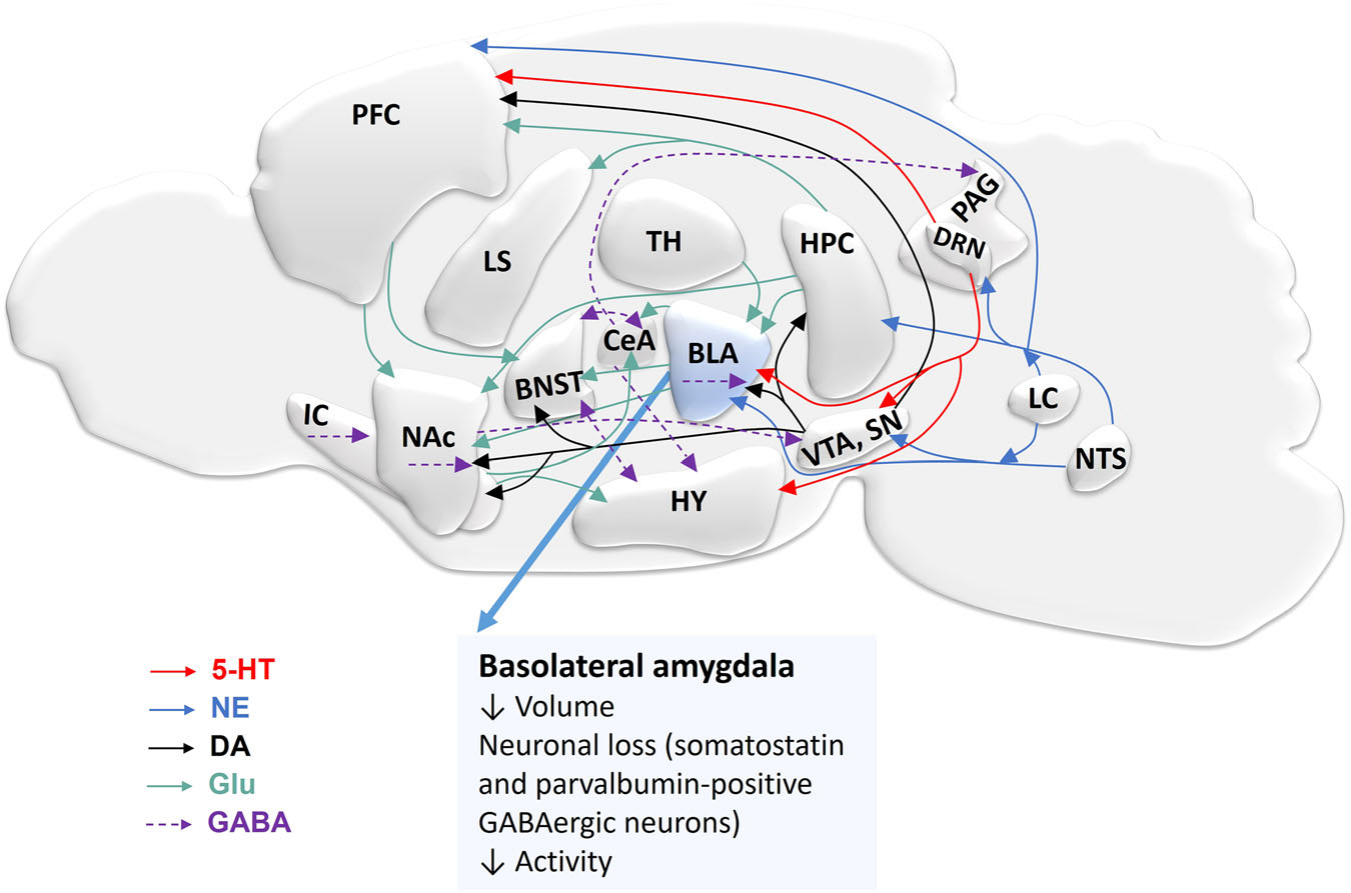
Overview of neuronal circuits involved in fear and anxiety in the rodent brain. The basolateral amygdala (BLA) is a central regulator of fear circuitry and other brain regions are involved in these processes. Interconnection of various brain regions in fear by different neurotransmitter pathways including serotonergic (red), glutamatergic (green), dopaminergic (black), norepinephrinergic (blue), and GABAergic (purple). Efferent projection of serotonin from dorsal raphe nucleus (DRN) to various brain regions such as BLA, ventral tegmental area (VTA), substantia nigra (SN), and prefrontal cortex (PFC). Glutamate projections interconnect different brain regions. Norepinephrine (NE) is released from projections of locus coeruleus (LC) and nucleus tractus solitarius (NTS). The VTA and SN provide dopaminergic inputs to the nucleus accumbens (NAc), bed nucleus of the stria terminalis (BNST), BLA, hippocampus (HPC), PFC, and insular cortex (IC). VTA and SN receive GABAergic projections from NAc and the central amygdala (CeA) sends GABAergic projections to the hypothalamus (HY) and the periaqueductal gray (PAG). BNST sends or receives GABAergic projection to/from HY and CeA; and GABAergic interneurons are present in NAc, IC, and BLA. Reduced amygdala volume, neuronal cell loss, and neuronal activity are associated with anxiety disorders. Serotonin: 5-hydroxytryptamine (5-HT); gamma-aminobutyric acid (GABA); dopamine (DA); glutamate (Glu); lateral septum (LS); thalamus (TH).

Previous work has suggested that fear is mediated by a brain-wide distributed network involving long-range projection pathways and local connectivity^13^. Thereby, the limbic system, such as the amygdala and the hippocampus, together with cortical regions play a critical role in fear response and anxiety (Fig. 1).

**Fig. 2:**
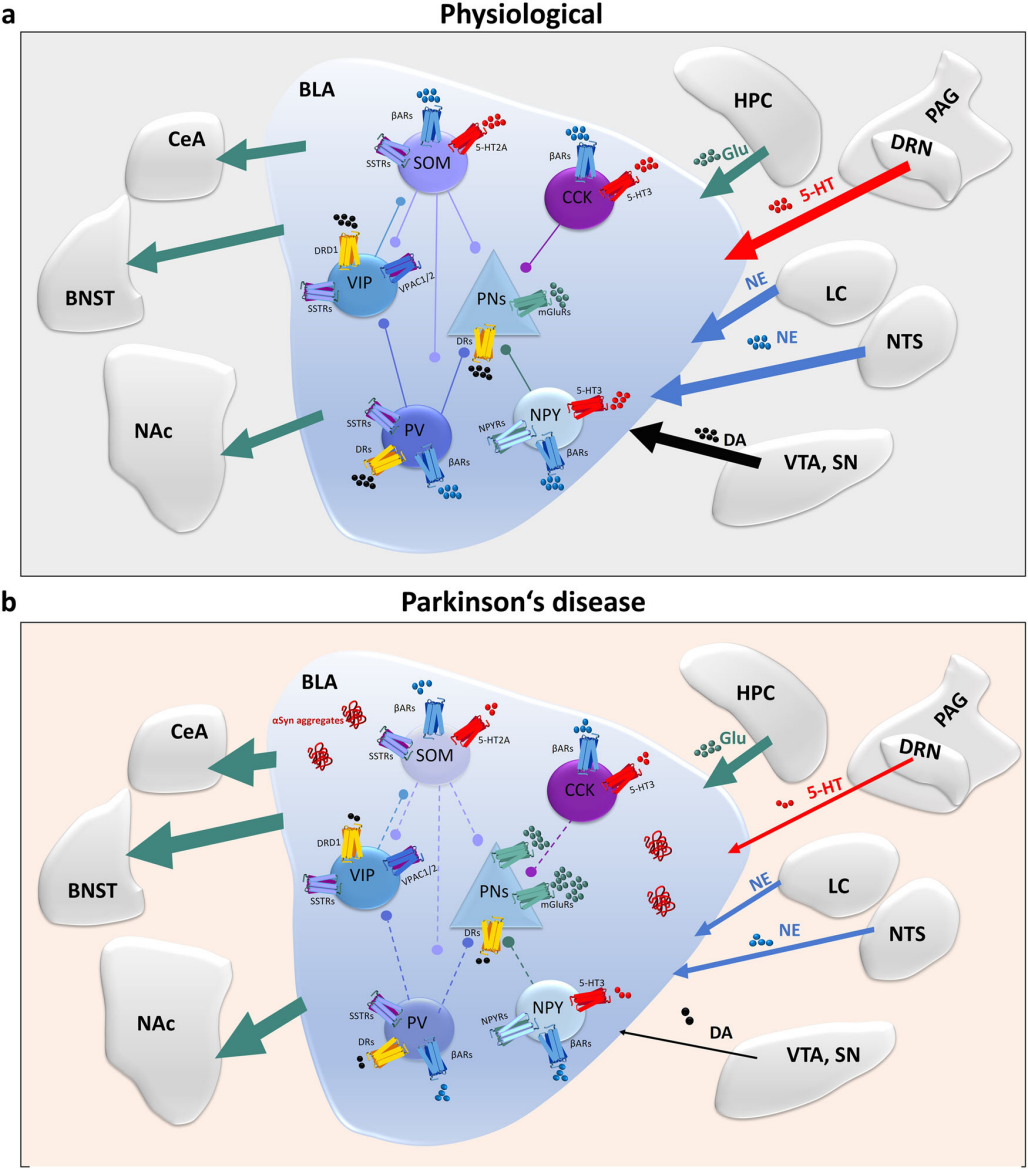
Schematic diagram illustrating the physiology and proposed pathophysiology of the neuronal circuitry in Parkinson’s disease. **a** Connectivity matrix among GABAergic neurons in the basolateral amygdala (BLA) and the major neural inputs/output to or from BLA. β-adrenergic receptors (βARs), serotonin receptors (5-HT3, 5-HT2A), dopamine receptors (DRD1 or DRs), somatostatin receptors (SSTRs), vasoactive intestinal polypeptide receptors (VPAC1/2) and metabotropic glutamate receptors (mGluRs) are expressed differently on interneurons. Interaction of principal neurons (PNs) and five subtypes of interneurons expressing somatostatin (SOM), vasoactive intestinal peptide (VIP), cholecystokinin (CCK), parvalbumin (PV), and neuropeptide Y (NPY) contributing to the regulation of anxiety. The excitatory output of PNs determines the fear response and is mainly reduced by the local interneuron network. PNs and interneurons receive multiple inputs: the serotonergic projection from DRN/PAG and glutamatergic projection from HPC, the NE projection from LC and NTS, and the dopaminergic projection from VTA and SN, while PNsreleases glutamate to NAc, BNST, and CeA. Depending on how PNs and interneurons are shifted in activity, these circuitries are involved in the regulation of fear response and memory. **b** Model for the pathophysiology of the neuronal circuitry of increased fear in PD. When αSyn aggregates are present, distinct activation patterns emerge in the glutamatergic (principal neurons) and GABAergic (5 types of interneurons) neurons in BLA. The inputs from VTA/SN, NTS, LC, DRN/PAG to BLA change. The reduction of dopamine produced from the VTA/SN pathway results in a decrease in a long-term depression on PNs^175^ but also in altered interconnections between interneurons expressing DRs. The decrease in norepinephrine and serotonin projection also contributes to changes in neuronal activity in the BLA^176^. In addition, interneurons could be more prone to neurotoxicity of αSyn aggregates^98^ leading to disinhibition of PNs. As a result, the output of glutamate increases (or increased glutamatergic activity and hyperexcitability) from BLA, triggering increased fear behavior^177,178^. Serotonin: 5-HT 5-hydroxytryptamine, GABA gamma-aminobutyric acid, DA dopamine, Glu glutamate, VTA ventral tegmental area, SN substantia nigra, LC locus coeruleus, PAG periaqueductal gray, DRN dorsal raphe nucleus, HPC hippocampus, NAc nucleus accumbens, BNST bed nucleus of the stria terminalis, CeA central amygdala.

The amygdala is a central region responsible for fear and anxiety^14^, as evident from studies of fear circuits in animals^6,15,16^. Consistent with these findings, functional imaging studies in humans have reported activation of the amygdala during conditioned fear acquisition and extinction^17,18^. The amygdala consists of multiple subdivisions such as the basolateral amygdala (BLA), the basomedial amygdala (BMA), the central amygdala (CeA), the medial amygdala (MeA), and the cortical amygdala (CoA), of which the BLA and CeA are particularly important in anxiety and fear processing^19,20^. It is accepted that an imminent danger activates the BLA, which by way of connections to the CeA initiates the expression of defensive behavioral reactions such as freezing together with physiological reactions^21^ (Fig. 2a). Importantly, microcircuits within the CeA are also crucial for fear extinction^11^. Furthermore, the hippocampal circuitry is implicated in both fear and anxiety^22–24^. Reductions in hippocampal and amygdalar volume have generally been observed in patients with anxiety^25,26^. Engin et al. recently demonstrated that inhibition of the principal neurons of the dentate gyrus and Cornu Ammonis 3 (CA3) region led to suppression of anxiety^23^. In addition, the direct projection from the ventral hippocampus to the medial prefrontal cortex is required for anxiety-related behaviors^27^. Several cortical and subcortical brain areas were shown to contribute^28–33^. Among those, the prefrontal cortex (PFC) is important for regulating the behavioral expression of fear and the extinction of previously acquired fear memories^29^. Also, the PFC plays a role in the initial formation of emotional memories involving sufficient temporal or contextual complexity^29^. The insular cortex activation was reported in fear and anxiety studies^31–34^. Recent findings indicate that the anterior agranular insular cortex was involved in the consolidation of fear memory^35^. Other brain regions such as the nucleus accumbens (NAc), the bed nucleus of stria terminalis (BNST), the periaqueductal gray (PAG), brain stem nuclei, thalamic nuclei, the superior colliculus, and the pulvinar contribute to the fear and anxiety circuitry^36–39^.

## Evidence for dysfunctions of anxiety and fear circuits in Parkinson’s disease and other synucleinopathy disorders

Parkinson’s disease (PD) is the second most common progressive neurodegenerative disorder, which is characterized by both motor and non-motor clinical features^40^. The presence of αSyn positive neuronal inclusions called Lewy bodies has been suggested to play a key role in the pathomechanisms of PD^40–42^. αSyn-associated pathology is thought to be involved in the loss of nigrostriatal dopaminergic neurons, which initiates the signature motor symptoms. Nevertheless, αSyn pathology is not exclusive to the dopaminergic neurons, and degeneration of other neuronal populations can be observed in different brain structures as well, leading to a plethora of non-motor symptoms^43,44^. The diagnosis of PD relies on the prominent expression of motor symptoms; however, by that time 50–70% of dopaminergic neurons are already lost. Interestingly, non-motor symptoms such as hyposmia, rapid eye movement sleep behavior disorder, mood disorders, and cognitive impairment can precede motor symptoms by several years^45,46^. Among the non-motor features, some types of anxiety are described in PD patients, such as generalized anxiety disorder (GAD), panic attacks, social phobia, phobic disorder, agoraphobia, and obsessive-compulsive disorder^47–49^. Based on current reports, anxiety has a prevalence ranging from 22.2% to 66.7% in synucleinopathy patients. Similar to the general population, female PD patients seem to be at higher risk for anxiety disorder compared to male patients^50^. However, there are studies reporting no increased risk in female PD patients^51^. Moreover, anxiety is present in both the on and off PD medication states^52–57^. Prevalence is increasing further as psychiatric symptoms are diagnosed more frequently as primary disease-related and not merely secondary to the disease burden.

### Prevalence of anxiety disorder in patients with Parkinson’s disease and other synucleinopathies (clinical evaluation)

As mentioned above, there is a high prevalence of anxiety disorder in PD patients^58^. In synucleinopathy patients, it appears challenging to discriminate anxiety from other common psychiatric manifestations such as depression and hallucination (PD, DLB, MSA). A thorough clinical assessment of anxiety-related symptoms is crucial for effective treatment. To this purpose, a reliable and validated clinical rating scale for assessing anxiety symptoms in patients with PD or other synucleinopathies is required. In the general population, anxiety disorders are typically classified according to the Diagnostic and Statistical Manual of Mental Disorders (DSM)^59^. The most common rating scale was DSM IV, and 34% of PD patients met its criteria for mental disorder^60^. Anxiety symptoms of PD patients are clinically evaluated by validated rating scales such as the recent disease-specific Parkinson’s anxiety scale (PAS)^49,61–65^. The PAS accesses different characteristics of anxiety such as persisting anxiety, episodic anxiety, or avoidance behavior^63,66^. These rating scales are also suitable to assess anxiety in other synucleinopathies disorders. Of note, neuroimaging has been recently employed to explore psychiatric disorders in patients including positron emission tomography (PET) and magnetic resonance imaging (MRI). Table 1 provides a summary of studies on the prevalence of anxiety in PD and other synucleinopathies. Interestingly, Upneja et al. reported that episodic anxiety was the most common anxiety subtype (50% of cases), while avoidance behavior and persistent anxiety were less common (35% and 15%, respectively)^63^. Of note, Moriyama et al. reported that 31% of PD patients presented with a social anxiety disorder (SAD), which is characterized by an abnormal fear and avoidance of scrutiny by others^67^. Anxiety also occurs frequently in patients with DLB or MSA (Table 1), but the anxiety rates vary among synucleinopathies. In DLB, more than 60% of patients show anxiety^68–70^, and anxiety symptoms can either decline^69^ or worsen over the course of the disease^70^. Of patients with MSA, over 50% have been diagnosed with anxiety^71–73^. This may relate to differences in underlying neuropathology such as different seeding properties of respective αSyn strains and affected cell types (oligodendrocytes accumulate αSyn positive-inclusions in MSA) and underlines that anxiety is not merely the result of the general disease burden^74^. In summary, anxiety is frequent in patients with PD/synucleinopathies but diagnosis varies with the applied anxiety rating scale, sex, population, age at disease onset, duration of disease, and Hoehn and Yahr (H&Y) stage. Moreover, some forms of anxiety in PD could be underreported by the employed anxiety rating scales, leading to underdiagnosed and undertreated symptoms^75^. A combination of several scales and the application of newly developed neuroimaging analyses described in the next chapter may improve diagnosis.

**Table 1:** Summary of anxiety findings in Parkinson’s disease and other synucleinopathy disorders.

Diagnosis	Age (years)	Average disease duration (years)	Subject number	Sex (males/females)	Country	Evaluation method	Main findings
PD	61.2 + 10.4	3.8 ± 3.5	105 PD	79/26	India	PAS	53.5% PD patients with anxiety^63^
	67.2 ± 10.6	n.a.	100 PD	61/39	Italy	PAS DSM-IV-TR	41% PD patients with anxiety 26% PD patients with anxiety^179^
	71.8 ± 10.7 69 ± 11.3	5.8 ± 3.2	64 PD 50 HC	33/31 26/24	Egypt	DSM-IV-TR and HAM-A	20% PD patients with anxiety^51^
	63.7 ± 8.9	10.9 ± 2.1	108 PD	72/36	United States	HAMA	66.7% PD patients with anxiety 55.5% PD patients with anxiety preceding PD diagnosis^76^ No significant differences in ages of onset regarding sex
	69.22 ± 7.53 71.64 ± 7.13	5.29 ± 3.76 (men) 5.64 ± 3.13 (women)	48 PD	32/16	Turkey	HAMA	62.8% of PD patients with anxiety^180^
	65.32 ± 5.18	5.78 ± 1.04	90 PD	47/ 43	China	HAMA	22.2% patients with possible anxiety 17.78% patients with definite anxiety^181^
	55 ± 13.5 63.8 ± 11.3	7.4 ± 3.9 (SAD) 7.8 ± 5.5 (without SAD)	110 PD	52/58	Brazil	DSM IV-TR	31% of patients with social anxiety disorder^67^
	66.1 ± 9.8	8.4 ± 6.9	123 PD	76/47	Netherlands	BAI	52.8% of patients with anxiety^182^
	64.5 ± 10.3	5.1	294 PD	178/116	Netherlands	BAI	45% of patients with anxiety^64^
	>51	n.a.	196 PD 196 HC	121/75 121/75	United States	DSM-IV Longitudinal study	Anxiety presents 5 years or more before the onset of motor symptoms^77^
	59.50 ± 9.4 60.68 ± 8.8	2.5	115 PD 78 HC	59/56 36/42	China	STAI	28.7% of patients with anxiety^183^
	61.7 60.8	6.65	423 PD 196 HC	277/146 126/70	International	STAI-State score STAI-Trait score	24.6% PD patients with anxiety vs. 7.7% anxiety in healthy controls^184^ 20.1% PD patients with anxiety vs. 9.7% anxiety in healthy controls^184^
DLB	76.7 ± 6.9	n.a.	41 DLB	17/24	Belgium	MMSE	63.4% of patients with anxiety^68^
	76 ± 7.3	n.a.	72 DLB	40/32	Norway	DSM-IV Longitudinal study	Anxiety declined over time^69^
	73 ± 7.4	n.a.	92 DLB	44/48	Italy	NPI	67.4% of patients with anxiety and anxiety symptoms worsened over the course of the disease^70^
MSA	59.63 ± 8.39	2.35 ± 1.21	237 MSA	111/126	China	HAMA	46.8% patients with mild anxiety 25.9% patients with moderate to severe anxiety^71^
	68.8 ± 6.9	6.3 ± 3.9	286 MSA	166/120	United Kingdom	HADS-A	54% of patients with anxiety^72^
	57.7 ± 7.3	3.3 ± 1.7	47 MSA	18/29	Serbia	HAMA	53% of patients with anxiety^73^

*PAS* Parkinson anxiety scale, *HAMA* Hamilton anxiety scale*, BAI* Beck anxiety inventory*, DSM-IV* diagnostic and statistical manual of mental disorders, fourth edition, *MMSE* Mini-mental state examination, *HADS-A* self-completed scale with anxiety, *STAI* State-Trait Anxiety Inventory.

Several studies suggest that anxiety is an early symptom of PD which appears to increase in incidence and severity with disease duration. In fact, anxiety may precede the cardinal PD motor symptoms in a majority of patients and anxiety disorders were described to manifest even decades prior to PD diagnosis^51,76,77^. Importantly, age at the onset of PD was reported to be younger in patients exhibiting anxiety^51,78^. Anxiety disorder patients were associated with a 38% increased risk of PD compared to those without anxiety during a 5.5-year follow-up period^61^. This and other data support that patients with anxiety disorders have a higher risk to develop PD^79,80^. A finding on a large and culturally heterogeneous sample indicates that the progression of PD patients with the H&Y stages 2–3 and 2–4 increased the likelihood of prevalence of anxiety^81^. This is in line with the previous study which found a positive correlation between anxiety and the severity of motor symptoms of PD^51,78^. More research is required to decipher whether this reflects the further progression of symptoms displayed early in the disease, which indicates ongoing underlying neuropathology (primary disease impact), or whether the increased disease burden causes additional anxiety symptoms (secondary disease impact). In fact, cognitive decline or co-pathology of tau and amyloid beta accumulation at advanced disease stages are likely to add (primary or secondary) to the development of anxiety symptoms or vice versa anxiety appears to predict the risk to develop mild cognitive deficits^82–84^. Of note, several studies showed that anxiety in PD is influenced by ON–OFF states of dopamine medication, motor fluctuations, and dyskinesia^52–57,75,78^. Some investigations reported increased anxiety symptoms in OFF-dopaminergic medication when lacking the beneficial effects of treatment on motor symptoms^52,53,85^. Pontone et al. recently identified that 31% in a total of 200 PD patients showed worse anxiety symptoms in the OFF-dopamine medication state compared to the ON-dopamine medication state^55^. In fact, few research groups also observed the improvement of anxiety under dopaminergic therapy^56,57^. In summary, representing an early symptom of disease, anxiety disorders may benefit from disease-modifying treatment. However, to develop rationale therapeutic strategies, it is important to understand the underlying pathophysiology of anxiety in synucleinopathies which is summarized in the next chapters.

### Structural and functional brain changes associated with anxiety in synucleinopathies

In recent years, neuroimaging analyses identified structural and functional changes associated with PD-related anxiety^86,87^. Functional MRI identified connectivity changes, such as increased limbic-orbitofrontal cortex, decreased limbic-dorsolateral prefrontal cortex and orbitofrontal-dorsolateral prefrontal cortex, and decreased sensorimotor-orbitofrontal cortex connectivity^88^. Reduced functional connectivity in the limbic cortico-striatal circuits and increased functional connectivity between the cerebellum and occipito-temporal regions were associated with a more impaired neuropsychiatric profile including anxiety in PD patients^89^. MRI of PD patients at the early stage of the disease showed that the severity of anxiety was associated with reduced structural covariance of the left striatal seeds with the contralateral caudate nucleus^90^. A recent study compared the proportion of spontaneous low-frequency fluctuations to the whole brain signal of resting-state functional MRI and found increased or decreased neuronal activities across multiple brain regions in PD patients with anxiety compared to PD patients without anxiety at early disease stages^91^. In drug-naive PD patients, abnormal intrinsic connectivity within and between large-scale networks may represent a potential neural correlate of anxiety symptoms even in the absence of clinically relevant cognitive impairment^92^. Furthermore, alterations in the amygdala, hippocampus, caudate nucleus, and anterior cingulate cortex are strongly suggested to underlie the development of anxiety in PD^26,93,94^. In high-density electroencephalography (hd-EEG) analysis, 31% of PD patients were displaying anxiety symptoms related to the prefrontal cortex^95^.

PD-associated anxiety is linked to structural changes in the amygdala (Figs. 1 and 2b) with dysfunction of different neuronal types which results in an excitation/inhibition shift^96–98^. Reduced amygdala volume and neuronal cell loss in post-mortem PD brains are associated with anxiety^99^. In line with this, a recent functional imaging study in 110 early-stage PD patients found that a smaller volume of the left amygdala is associated with anxiety symptoms^26^. These studies are consistent with previous findings in anxiety patients without PD^25,100–102^. Interestingly, idiopathic rapid eye movement sleep behavior disorder (iRBD) patients, which frequently develop PD, showed reduced gray matter volume in the left amygdala extending to the hippocampus. Importantly, this was present only in iRBD patients with anxiety, not in healthy controls or iRBD patients without anxiety^103^. This demonstrated the occurrence of anxiety together with structural alterations in limbic brain regions prior to cardinal motor symptoms and PD diagnosis. Importantly, the appearance of αSyn pathology in the amygdala, leading to a significant decrease of somatostatin and parvalbumin-positive interneurons, has been observed in PD patients^96^. These neuronal populations in the amygdala may therefore represent structural correlates and harbor pathophysiological mechanisms driving anxiety in PD.

Abnormal activity and connectivity of the amygdala can also be related to neurochemical alterations in PD patients with anxiety^94^ (Fig. 2b). Reduction of dopamine in the caudate putamen is a hallmark of PD accounting for the cardinal motor symptoms, but the dysregulation of the dopaminergic system is also linked to non-motor symptoms of PD. Whether dopamine plays a critical role in originating anxiety in PD is poorly understood. Reduced dopamine transporter (DAT) in the striatum of PD patients correlates with anxiety^104,105^. In experimental models, dopaminergic projections from the ventral tegmental area (VTA), which has long been suggested to be involved in PD, to the basal amygdala modulate fear and anxiety^106–108^.

Furthermore, disturbances of the serotonergic and cholinergic systems were reported in PD patients, possibly involved in non-motor symptoms^109–111^. Serotonin (5-HT) is prominently expressed in the amygdala^112^ and other limbic areas, and alterations of the serotonergic system were observed in the early stage of PD^109^. Both cholinergic and serotonin receptors were significantly altered in the putamen post-mortem^111^. Identification of genetic variants in the serotonin transporter (SERT) gene promoter regions showed that PD patients with the short allele of the serotonin transporter promoter scored significantly higher on anxiety measures^113^. Higher levels of anxiety in PD patients are associated with lower thalamic SER in PET studies, pointing towards a contribution of serotonergic degeneration to anxiety symptoms in PD^114^. In addition, serotonin levels appear reduced in the brain of PD patients^115^. Lastly, alterations of gamma-aminobutyric acid (GABA) and norepinephrine (NE) systems have also been implied in the development of anxiety in PD^48^.

## Insights from animal models of synucleinopathy regarding fear and anxiety

If PD-related neuropathology is underlying the development of anxiety, effective symptomatic or ideally disease-modifying treatment requires knowledge of the pathomechanisms and cellular substrates driving these symptoms. Pavlovian fear conditioning, elevated plus maze, open field test, and the burying behavior test have been applied in models of anxiety disorder in PD. Altogether, these studies confirmed the involvement of catecholamine and GABAergic neuronal circuitry in this phenotype^116–118^. Notably, the contribution of αSyn-associated pathology in limbic brain regions was also found to associate with anxiety and fear symptoms in animal models of PD. Interestingly, studies in anxiety models (unrelated to PD) proposed an increase of hippocampal αSyn expression under high levels of innate anxiety, possibly mediated via dopaminergic mechanisms^119^. This may indicate that anxiety can drive αSyn accumulation leading to a feed-forward loop. Thus, it remains to be clarified whether anxiety is a bystander or a participant in synucleinopathy progression. Such mechanisms require urgent attention, as this would indicate that early therapeutic intervention to reduce fear and anxiety in PD could be disease-modifying for other progressive neuropathologies in PD. The following sections and Table 2 summarize the anxiety and fear-related findings from animal models of PD, with special emphasis on druggable molecular mechanisms and the role of αSyn. For further details and general remarks on animal models of PD, see reviews in refs. ^120–122^.

**Table 2:** Summary of anxiety findings in synucleinopathy animal models.

Animal model	Behavior test	Time	Non-motor feature findings vs controls	Mechanism findings
6-OHDA	OFT, EPM	3 weeks	No effect	n.a.^123^
	OFT, EPM	36 days	Anxiety	n.a.^124^
	EPM	3 weeks	Anxiety	n.a.^117,126^
	EPM Contextual fear conditioning	21 days 24 days	Anxiety	Amygdala: reduced dopamine, norepinephrine, increased serotonin Striatum: reduced dopamine, norepinephrine, serotonin Prefrontal cortex: reduced dopamine, norepinephrine, serotonin^116^
	Marble burying OFT, EPM	3 weeks	Anxiety	Catecholaminergic denervation^127^
	OFT, EPM	3 weeks	Anxiety	Activation or suppression of AMPA receptor in the lateral habenula led to increase or decrease anxiety, respectively^128^
MPTP	EPM	26 days	Anxiety	Whole brain lysate: reduced serotonin, dopamine^125^
	EPM	5 days	Anxiety	n.a.^131^
Rotenone	EPM, OFT	28 days	Anxiety	Prefrontal cortex, hippocampus: reduced 5-HT+cell and SERT+cell density BLA: reduced 5-HT+cell density^134^
Paraquat	EPM	1 month	Anxiety	n.a.^136^
PFF	OFT	6 months	No effect	n.a.^139^
	Fear conditioning	3, 6 months	No effect	n.a.^144^
	OFT EPM	30 days	Anxiety	Highest level of αSyn in the amygdala^143^
	OFT Elevated zero maze Cued and contextual fear conditioning	6 months	Anxiety	Amygdala: 18% neuronal loss, αSyn inclusion colocalized with excitatory neurons Prefrontal cortex: no neuronal loss^142^
	Fear conditioning Contextual fear retention	9 months	Anxiety	n.a.^150^
A53T	EPM	12 months	Reduced anxiety	n.a.^153^
	OFT Stress-induced hyperthermic	2, 6, 12 months	Reduced anxiety	n.a.^185^
	Open field	12 months	Increased anxiety	n.a.^161^
	EPM	2, 8, 12 months	Reduced anxiety	Striatum: no change in NE, serotonin, reduced DAT at 8 months^145^
	OFT, EPM	12, 24 weeks	Reduced anxiety	Striatum: DAT levels significantly decreased and increased dopamine level and its metabolites DOPA^146^ Hypothalamus: increased serotonin levels^146^
	EPM	3, 5, 7 months	Reduced anxiety	Hypothalamus and hippocampus: αSyn inclusion colocalized with orexin neurons^158^ Activation of orexin neurons restores anxiety^158^
A30P	Fear conditioning	4, 12 months	Reduced freezing	n.a.^147^
BCA αSyn	EPM	12, 24 months	Reduced anxiety	Striatum: Increased DAT and serotonin^148^
	Exploratory and feeding behavior	4 months	Anxiety	Hippocampus: reduced serotonin^149^
A53T αSyn/Tau KO	OFT	2, 6, 12, 18 months	Anxiety	SN: loss of parvalbumin neurons^159^
Thy1-αSyn	Fear conditioning	3, 4 months	Increased fear	Amygdala: loss of parvalbumin neurons, microgliosis^98^
αSyn KO	EPM	12 weeks, 12 months	No effect	n.a.^153,154^
LRRK2	EPM	4, 20 months	No effect	n.a.^166^
Dj-1 KO	EPM	8, 17 months	No effect	n.a.^186^
	EPM	3, 5 months	Anxiety	Anxiety-like behaviours were substantially reversed by modulating D2 receptor activity^168^
Park2 KO	EPM	n.a	Anxiety	n.a.^169^
Pink1 KO	EPM	4, 8, 12 months	Anxiety	Locus coeruleus: reduced number of cell bodies immunoreactive for TH, and increased expression of the alpha -1 adrenergic receptor^170,171^

*MFB* medial forebrain bundle, *n.a.* not applicable, *DAT* dopamine transporter, *EPM* elevated plus maze, *OPT* open field test, *SN* substantia nigra, *NE* norepinephrine, *DOPA* 3,4-dihydroxyphenylalanine, TH tyrosine hydroxylase, *AMPA* aminomethylphosphonic acid.

### Toxic-based animal models of PD

Neurotoxin 6-hydroxydopamine (6-OHDA) is a toxin widely used to degenerate dopaminergic neurons in the substantia nigra and thereby cause the loss of striatal dopamine leading to robust motor symptoms. Initially, anxiety features were not detected in the 6-OHDA animal models^123^. However, recent findings indicated anxiety-like behavior using the elevated plus maze, the fear conditioning test, or the open field test, among others^116,117,124,125^. Dopaminergic innervation in the nigrostriatal pathway or the globus pallidus led to anxiety-like behavior^125,126^. Interestingly, a significant increase of serotonin was observed in the amygdala of 6-OHDA lesioned rats, while dopamine and norepinephrine (NE) were reduced. In contrast, NE and 5-HT significantly decreased in the prefrontal cortex and striatum. The data suggest that the alterations of these neurotransmitters in either amygdala, prefrontal cortex, or striatum result in anxiety-like behavior^116^. 5-HT_7_ receptors in the prelimbic cortex or 5-HT_6_ receptors in the dorsal hippocampus appear involved in the regulation of anxiety-like behaviors in 6-OHDA lesioned rodents related to changes in dopamine, serotonin, and NE levels^117,118^.

Interestingly, intra-amygdalar 6-OHDA injections to produce targeted catecholaminergic denervation led to the loss of tyrosine hydroxylase positive (TH+) fibers in the amygdaloid complex, in the ventral part of amygdalo-striatal transition zone and reduced number of TH+ neurons in the substantia nigra compacta (SNc) and VTA. As a result, increased anxiety-like behavior was observed, which suggests that loss of catecholaminergic neurotransmission in the amygdala may contribute to anxiety symptoms in PD^127^. Interestingly, a recent study proposes that the lateral habenula (a crucial structure of the epithalamus and neuronal axons from the lateral habenula project onto monoaminergic neurons in the midbrain) is highly involved in the regulation of anxiety-like behavior^128^. It should be kept in mind, however, 6-OHDA does not cross the blood–brain–barrier and must be surgically microinjected in selected brain regions, to induce rapid toxicity with immediate onset of impairment in the dopamine system that affects anxiety detection and pathophysiology of anxiety^129^. Despite the limitation, the 6-OHDA model was successful in generating anxiety-like behavior and is useful to study how targeted lesions of catecholaminergic neurons contribute to this phenotype (face validity).

Other toxins well-established to produce dopamine neuron loss in PD models include systemic application of 1-methyl-4-phenyl-1,2,3,6-tetrahydropyridine (MPTP), rotenone, and paraquat^130^. Mostly, studies adopting the use of those toxins have demonstrated motor, cognitive, mitochondrial dysfunction, neurochemical, and molecular changes. Recently, non-motor symptoms, especially an anxiety-like phenotype were detected in a few studies^131–136^. For instance, the increased anxiety level was found in the elevated plus maze (EPM) test of MPTP-injected animals compared to the control group^131,132,137^. In the rotenone model, Tsarouchi et al. reported the correlation of altered 5-HT and serotonin transporter (SERT) immunodensity levels in the cortico-limbic system with increased anxiety-like phenotype in the open field test (OFT) and EPM^134^. Increased anxiety-like behaviors were also reported in paraquat-exposed rats^136^.

Overall, the neurotoxin models highlight that some aspects of anxiety in PD could be associated with similar neurochemical changes as motor symptoms in specific brain regions. However, the connection to synucleinopathy is lacking since αSyn is not directly involved in these models. Thus, these models are limited with regard to reflecting disease pathogenesis and mechanisms, which underlie neurodegeneration (construct validity) but may be sufficient to develop symptomatic therapy, especially for symptoms related to late PD stages (predictive validity).

### Genetic animal models of PD

αSyn accumulation in Lewy bodies represents a hallmark of PD and αSyn-related pathology is the signature of synucleinopathies. There is a lack of knowledge on the role of αSyn pathology in the development of anxiety and fear in PD. Very few studies using either adeno-associated virus-αSyn models or pre-formed-fibril (PFF)-induced models have evaluated the association between αSyn pathology and the development of non-motor symptoms of PD, with rare attention to anxiety-related behaviors^138–142^. Intracerebroventricular injection of αSyn oligomers induced increased anxiety-like behavior compared to vehicle or αSyn monomers-injected animals at 20 days post-injection (dpi) in the elevated plus maze test^139^. Burtscher et al. observed anxiety-like behaviors in the elevated plus maze of PFF-injected mice at 30 dpi, but the fear conditioning test showed no difference compared to vehicle-injected mice^143^. This study also indicated that the amygdala is preferentially affected by αSyn accumulation, but most behaviors associated with the amygdala such as fear were unaffected^143^. Thus, αSyn accumulation in this model did not immediately impair amygdala physiology. In another study, the authors also did not detect any difference in the acquisition of fear, or in contextual or cued recall of PFF-injected mice compared to the control group receiving monomeric αSyn injection after 3 months post motor cortex injection^144^. However, Stoyka et al. recently showed a reduction in fear conditioning in intrastriatal PFF-injected mice^142^ probably related to αSyn pathology-induced loss of excitatory neurons in the amygdala.

Similarly, decreased anxiety-like behavior was reported in transgenic mice overexpressing human αSyn with a PD-associated mutation (A53T)^145,146^. Also, reduced freezing in Pavlovian fear conditioning was shown in transgenic mice with another PD-associated αSyn mutation (A30P)^147^. Similarly, αSyn transgenic mice under the endogenous promotor showed a decrease in anxiety-like behavior^148,149^. In contrast and more relevant to increased anxiety in PD patients, only one study on A53T transgenic mice showed higher anxiety-like behaviors in open field tasks and the elevated plus maze^107^. A recent study reported that αSyn pathology transmitted from the olfactory bulb induced anxiety-like behavior in αSyn PFF injected αSyn transgenic mice^150^. Recently, we demonstrated enhancement of fear responses in the Pavlovian fear conditioning in mice overexpressing human wild-type alpha-synuclein (Thy1-αSyn, line 61), an established and extensively characterized model of PD^98,151,152^. This phenotype does not represent a loss of function of αSyn, as αSyn KO mice do not show an anxiety phenotype in the open field or elevated plus maze tests^153,154^.

Regarding mechanisms, numerous studies in the transgenic αSyn models supported a contribution of dysregulated serotonergic signaling in anxiety^149,155,156^. As mentioned above, PD patients with anxiety show dysfunction in several neurotransmitter systems. αSyn has been shown to accumulate in serotonergic neurons^155^. Even though there was no loss of serotonergic neurons in the dorsal and median raphe nuclei of 1-year-old αSyn A53T transgenic mice, serotonin levels in the brain stem and serotonergic fiber density in the dorsal dentate gyrus were reduced^155^. In line with this, the accumulation of human αSyn resulted in an early deficit of the serotonergic system which was associated with an anxiety-like phenotype in the transgenic rat model of PD^107^. These data suggest that toxicity of αSyn may lead to dysfunction of serotonergic neurons, which is linked to anxiety induction in PD animal models. Other monoaminergic neurons may be involved as well: a recently developed transgenic mouse model expressing human αSyn only in noradrenergic neurons displayed anxiety-like behavior in the marble-burying test at 14 and 24 months of age^157^.

Interestingly, Stanojlovic et al. recently showed that chemogenetic activation of orexin neurons in A53T mice restores the deficits in anxiety-like behavior^158^. Recent findings suggest that tau knockout can exacerbate A53T αSyn-induced loss of parvalbumin-positive neurons in substantia nigra pars reticulata (SNR), which is accompanied by an exhibition of anxiety-like behavior^159^. Also, reduced GABAergic parvalbumin-positive neurons were observed in the amygdala of Thy1-αSyn mice with increased fear phenotype^98^. In addition, changes in fear response were accompanied by astrogliosis and microgliosis in the A53T model and Thy1-αSyn mice^98,158^. This lends support to the role of inflammation in anxiety-related disorders, which was discussed recently^160^.

In summary, these models provide some evidence for a link between αSyn-related pathology and deficits or enhancement of anxiety-like behavior, but as with every model these models also have limitations. While there are some studies that report increased anxiety in A53T models^161–163^, the decreased anxiety phenotype appears prominent in A53T transgenic mice yet incongruous with typical clinical symptoms, while Thy1-αSyn mice show a phenotype consistent with increased anxiety in PD. This model could be useful to decipher the underlying pathophysiological mechanisms thereby guiding improved and more rational therapeutic intervention. These contradictory observations in animal models may be due to differences between mutated versus physiological αSyn overexpression, promotor selection, and how the testing was performed, among others. Many αSyn-based models do not develop progressive neurodegeneration of dopaminergic neurons and some do not even display clear motor symptoms^120,164,165^. Thus, these models are less useful to study anxiety at advanced PD stages and the effects of dopamine therapy. In Thy1-aSyn mice the anxiety phenotype is developed prior to overt dopamine loss at 14 months of age and does therefore not reflect anxiety under dopamine depletion. Interestingly, mice display increased extracellular dopamine at an age when anxiety symptoms occur, together with fine motor deficits, cognitive dysfunction, hyperactivity, and αSyn pathology in the limbic and nigrostriatal system^152^. In animal models with complex behavioral phenotypes, anxiety may also develop as the response of the animal to the impairments in other modalities. Thus anxiety and fear phenotyping should cover a complex battery of tests, including components that do not depend on motor function. Hence, if used carefully, αSyn-related models can be useful to understand how αSyn related pathology in specific brain regions contributes to anxiety phenotypes and whether disease-modifying therapeutic strategies may ameliorate these symptoms.

In addition to transgenic αSyn animal models, some studies also explored the anxiety-like behavior in different transgenic models of early-onset PD such as LRRK2, PINK1, and DJ-1. LRRK2 transgenic mice represent the most common genetic cause of sporadic and familial PD but did not display anxiety-like behavior with age^166^. In line with this, DJ-1 KO rats or parkin-deficient mice do not develop anxiety-like behaviors^167^. In contrast, anxiety phenotypes were exhibited by DJ-1 KO mice^168^ as well as by Pink1 KO and Park2 KO mice^169^ or Pink1 KO rats^170,171^.

## Therapeutic implications

There is no treatment to stop or halt the development of anxiety in PD patients, as there is no cure or intervention that interferes with neuronal loss in synucleinopathies. Table 3 summarizes the current therapeutic options. Given the negative impact on the quality of life of patients, rational treatment of anxiety-related symptoms should receive greater attention. Among the currently used symptomatic interventions, antidepressant medications consist of selective serotonin reuptake inhibitors (SSRIs), serotonin-norepinephrine reuptake inhibitors (SNRIs), and tricyclic antidepressants (TCAs), but their efficacy in PD is insufficient. Also, benzodiazepines are frequently used to improve anxiety symptoms in PD patients, but they can trigger unfavorable side effects for patients such as falls and cognitive slowing^172^. Given the potential involvement of dopamine loss in the development of anxiety in PD, dopamine agonists may represent an alternative. Importantly, non-pharmacological interventions are frequently recommended. For example, physical activities can reduce anxiety. A recent study on Brazilian patients with PD showed a significant reduction in anxiety levels after exercise^173^. Alternatively, mindfulness yoga was effective in reducing anxiety in PD patients^174^. Preclinical and clinical trials which target the modulation of different neurotransmitter systems to reduce anxiety in PD are ongoing and may open up future directions.

**Table 3:** Therapeutic implications of PD on anxiety-related disorders.

Target	Compound	Description/mechanism	Status
GABA-A receptors agonist	Benzodiazepines	Binding to the type A gamma-aminobutyric acid receptors and potentiating inhibitory neurotransmission	Clinical^172^
5-HT receptor type 1A agonist	Buspirone	Decrease the activation of the 5-HT receptor	Clinical
SSRIs	Citalopram	Inhibit neuronal reuptake of serotonin in the synaptic cleft	Clinical
	Sertraline	Selectively inhibits the reuptake of serotonin at the presynaptic neuronal membrane, thereby increasing serotonergic activity	Clinical
NSRIs	Venlafaxine	Inhibit the reuptake of both serotonin and norepinephrine with a potency greater for the 5-HT than for the NE reuptake process	Clinical
Tricyclic antidepressant	TCA	Increase the synaptic level of NE and 5-HT by blocking their reuptake	Clinical
Dopamine agonist	Rotigotine transdermal patch	No results available	Phase 4 NCT02365870
	Psilocybin therapy	Recruiting, no results available	Phase 2 NCT04932434
	Buspirone	No results available	Phase 2 NCT02803749
Noradrenaline reuptake inhibitor	Atomoxetine	Reduction of anxiety-like behavior in PINK1 KO mice	Preclinical^171^
Other	Silibinin	Improved anxious-like behaviors in MPTP-induced PD	Preclinical^118^
	Multi-strain probiotic (TAP)	No results available	Phase 2 NCT03968133
	Acupuncture	Not yet recruiting	Clinical Trial NCT04729010
	Acupuncture	Real acupuncture significant ameliorate anxiety in PD patients at 2 months after treatment but sham acupuncture did not	Chinese Clinical Trial ChiCTR2100047253

*5-HT* serotonin, *NE* norepinephrine, *TCA* tricyclic antidepressant, *MPTP* 1-methyl-4-phenyl-1,2,3,6-tetrahydropyridine, *PD* Parkinson’s disease.

## Conclusion

While anxiety in PD patients has long been interpreted as resulting from debilitating motor symptoms and the loss in quality of life caused by the disease, it is now accepted as an early symptom, which reflects pathology in cortico-limbic systems and the corresponding neuro-circuitries and transmitter systems. In fact, the progressive development of fear and anxiety early in disease pathogenesis may represent a driver of pathology, for example by corresponding upregulation of αSyn pathology in limbic brain regions, or by over-excitation of affected neurons. In DLB early pathology in the limbic system is established, and the recent evidence from imaging studies and re-evaluation of neuropathology described in this review suggest early involvement of the BLA pathology in PD. A better understanding of the involved brain regions, neurons, and signaling pathways could provide novel therapeutic avenues. If αSyn-related pathology is a central mechanism to dysfunction in the cortico-limbic system, then αSyn targeted therapeutics could prove disease-modifying for these non-motor symptoms as well, which requires more attention.

### Reporting summary

Further information on research design is available in the Nature Research Reporting Summary linked to this article.

## Supplementary information

Reporting Summary

## References

[1] Perusini JN, Fanselow MS (2015). Neurobehavioral perspectives on the distinction between fear and anxiety. Learn. Mem..

[2] Babaev O, Piletti Chatain C, Krueger-Burg D (2018). Inhibition in the amygdala anxiety circuitry. Exp. Mol. Med..

[3] Calhoon GG, Tye KM (2015). Resolving the neural circuits of anxiety. Nat. Neurosci..

[4] Steimer T (2022). The biology of fear- and anxiety-related behaviors. Dialogues Clin. Neurosci..

[5] Craig, K. J., Brown, K. J. & Baum, A. Environmental factors in the etiology of anxiety. In *Psychopharmacology: The Fourth Generation of Progress* (eds Bloom, F. E. & Kupfer D. J.) 1325–1339 (Raven Press, New York, NY. 1995).

[6] LeDoux JE, Pine DS (2016). Using neuroscience to help understand fear and anxiety: a two-system framework. Am. J. Psychiatry.

[7] Penninx BW, Pine DS, Holmes EA, Reif A (2021). Anxiety disorders. Lancet.

[8] Baxter AJ, Scott KM, Vos T, Whiteford HA (2013). Global prevalence of anxiety disorders: a systematic review and meta-regression. Psychol. Med..

[9] LeDoux J (1998). Fear and the brain: where have we been, and where are we going?. Biol. Psychiatry.

[10] Shechner T, Hong M, Britton JC, Pine DS, Fox NA (2014). Fear conditioning and extinction across development: evidence from human studies and animal models. Biol. Psychol..

[11] Whittle N (2021). Central amygdala micro-circuits mediate fear extinction. Nat. Commun..

[12] Bystritsky A, Khalsa SS, Cameron ME, Schiffman J (2013). Current diagnosis and treatment of anxiety disorders. P T..

[13] Tovote P, Fadok JP, Luthi A (2015). Neuronal circuits for fear and anxiety. Nat. Rev. Neurosci..

[14] Han JS, McMahan RW, Holland P, Gallagher M (1997). The role of an amygdalo-nigrostriatal pathway in associative learning. J. Neurosci..

[15] Zelikowsky M, Hersman S, Chawla MK, Barnes CA, Fanselow MS (2014). Neuronal ensembles in amygdala, hippocampus, and prefrontal cortex track differential components of contextual fear. J. Neurosci..

[16] Lang PJ, Davis M, Ohman A (2000). Fear and anxiety: animal models and human cognitive psychophysiology. J. Affect Disord..

[17] LaBar KS, Gatenby JC, Gore JC, LeDoux JE, Phelps EA (1998). Human amygdala activation during conditioned fear acquisition and extinction: a mixed-trial fMRI study. Neuron.

[18] Agren T (2012). Disruption of reconsolidation erases a fear memory trace in the human amygdala. Science.

[19] Beyeler, A. & Dabrowska, J. Neuronal diversity of the amygdala and the bed nucleus of the stria terminalis. In *Handbook of Amygdala Structure and Function Handbook of Behavioral Neuroscience* Vol. 26 (eds Urban, J. H. & Rosenkranz, J. A.) 63–100 (Elsevier, 2020) Epub 31 Mar 2020. 10.1016/b978-0-12-815134-1.00003-9. 10.1016/b978-0-12-815134-1.00003-9 PMC7423190 32792868

[20] Janak PH, Tye KM (2015). From circuits to behaviour in the amygdala. Nature.

[21] Kopchia KL, Altman HJ, Commissaris RL (1992). Effects of lesions of the central nucleus of the amygdala on anxiety-like behaviors in the rat. Pharmacol Biochem Behav..

[22] Sanders MJ, Wiltgen BJ, Fanselow MS (2003). The place of the hippocampus in fear conditioning. Eur. J. Pharmacol..

[23] Engin E (2016). Modulation of anxiety and fear via distinct intrahippocampal circuits. Elife.

[24] Caliskan G, Stork O (2019). Hippocampal network oscillations at the interplay between innate anxiety and learned fear. Psychopharmacology (Berlin).

[25] van Mierlo TJ, Chung C, Foncke EM, Berendse HW, van den Heuvel OA (2015). Depressive symptoms in Parkinson’s disease are related to decreased hippocampus and amygdala volume. Mov. Disord..

[26] Vriend C (2016). A smaller amygdala is associated with anxiety in Parkinson’s disease: a combined FreeSurfer-VBM study. J. Neurol. Neurosurg. Psychiatry.

[27] Padilla-Coreano N (2016). Direct ventral hippocampal-prefrontal input is required for anxiety-related neural activity and behavior. Neuron.

[28] Myers-Schulz B, Koenigs M (2012). Functional anatomy of ventromedial prefrontal cortex: implications for mood and anxiety disorders. Mol. Psychiatry.

[29] Gilmartin MR, Balderston NL, Helmstetter FJ (2014). Prefrontal cortical regulation of fear learning. Trends Neurosci..

[30] Rozeske RR, Valerio S, Chaudun F, Herry C (2015). Prefrontal neuronal circuits of contextual fear conditioning. Genes Brain Behav..

[31] Alvarez RP, Biggs A, Chen G, Pine DS, Grillon C (2008). Contextual fear conditioning in humans: cortical-hippocampal and amygdala contributions. J. Neurosci..

[32] Shin LM, Liberzon I (2010). The neurocircuitry of fear, stress, and anxiety disorders. Neuropsychopharmacology.

[33] Gogolla N (2017). The insular cortex. Curr. Biol..

[34] Terasawa Y, Shibata M, Moriguchi Y, Umeda S (2013). Anterior insular cortex mediates bodily sensibility and social anxiety. Soc. Cogn. Affect. Neurosci..

[35] Shi T, Feng S, Wei M, Zhou W (2020). Role of the anterior agranular insular cortex in the modulation of fear and anxiety. Brain Res. Bull..

[36] Ray MH, Russ AN, Walker RA, McDannald MA (2020). The nucleus accumbens core is necessary to scale fear to degree of threat. J. Neurosci..

[37] Kim EJ (2013). Dorsal periaqueductal gray-amygdala pathway conveys both innate and learned fear responses in rats. Proc. Natl Acad. Sci. USA.

[38] Penzo MA (2015). The paraventricular thalamus controls a central amygdala fear circuit. Nature.

[39] Ren C, Tao Q (2020). Neural circuits underlying innate fear. Adv. Exp. Med. Biol..

[40] Kouli, A., Torsney, K. M. & Kuan, W. L. Parkinson’s Disease: Etiology, Neuropathology, and Pathogenesis. In *Parkinson’s**Disease: Pathogenesis and Clinical Aspects [Internet]*. (eds Stoker, T. B. & Greenland, J. C.) Brisbane (AU): Codon Publications. Chapter 1 (2018). 30702842

[41] Stefanis L (2012). alpha-Synuclein in Parkinson’s disease. Cold Spring Harb. Perspect. Med..

[42] Spillantini MG, Crowther RA, Jakes R, Hasegawa M, Goedert M (1998). alpha-Synuclein in filamentous inclusions of Lewy bodies from Parkinson’s disease and dementia with Lewy bodies. Proc. Natl Acad. Sci. USA.

[43] Maiti P, Manna J, Dunbar GL (2017). Current understanding of the molecular mechanisms in Parkinson’s disease: targets for potential treatments. Transl. Neurodegener..

[44] Yamazaki M (2000). Alpha-synuclein inclusions in amygdala in the brains of patients with the parkinsonism-dementia complex of Guam. J. Neuropathol. Exp. Neurol..

[45] Schapira AHV, Chaudhuri KR, Jenner P (2017). Non-motor features of Parkinson disease. Nat. Rev. Neurosci..

[46] Jellinger KA (2017). Neuropathology of nonmotor symptoms of Parkinson’s disease. Int. Rev. Neurobiol..

[47] Marsh L (2009). Anxiety disorders in Parkinson’s disease. Int. Rev. Psychiatry.

[48] Simuni, T. & Fernandez, H. H. Anxiety in Parkinson’s Disease. In *Parkinson’s Disease and Nonmotor Dysfunction* (eds Pfeiffer, R. F. & Bodis-Wollner, I.) *Curr. Clin. Neurol*. Ch. 2, 17–29 (Humana Press, Totowa, NJ. 2013). 10.1007/978-1-60761-429-6_2.

[49] Leentjens AF (2008). Anxiety rating scales in Parkinson’s disease: critique and recommendations. Mov. Disord..

[50] Dissanayaka NNW (2015). Characteristics and treatment of anxiety disorders in Parkinson’s disease. Mov. Disord. Clin. Pract..

[51] Khedr, E. M., Abdelrahman, A. A., Elserogy, Y., Zaki, A. F. & Gamea, A. Depression and anxiety among patients with Parkinson’s disease: frequency, risk factors, and impact on quality of life. Egypt. J. Neurol. Psychiatry Neurosurg. **56**, 10.1186/s41983-020-00253-5 (2020).

[52] Ganjavi H, MacDonald PA (2015). ON–OFF effects of dopaminergic therapy on psychiatric symptoms in Parkinson’s disease. J. Neuropsychiatry Clin. Neurosci..

[53] Racette BA (2002). Clinical features and comorbidity of mood fluctuations in Parkinson’s disease. J. Neuropsychiatry Clin. Neurosci..

[54] Rabinak CA, Nirenberg MJ (2010). Dopamine agonist withdrawal syndrome in Parkinson disease. Arch. Neurol..

[55] Pontone GM (2022). ‘Anxious fluctuators’ a subgroup of Parkinson’s disease with high anxiety and problematic on-off fluctuations. Parkinsonism Relat. Disord..

[56] Maricle RA, Nutt JG, Valentine RJ, Carter JH (1995). Dose-response relationship of levodopa with mood and anxiety in fluctuating Parkinson’s disease: a double-blind, placebo-controlled study. Neurology.

[57] Stacy MA, Murck H, Kroenke K (2010). Responsiveness of motor and nonmotor symptoms of Parkinson disease to dopaminergic therapy. Prog. Neuro-Psychopharmacol. Biol. Psychiatry.

[58] Broen MP, Narayen NE, Kuijf ML, Dissanayaka NN, Leentjens AF (2016). Prevalence of anxiety in Parkinson’s disease: a systematic review and meta-analysis. Mov. Disord..

[59] Broen MPG (2018). Clinical markers of anxiety subtypes in Parkinson disease. J. Geriatr. Psychiatry Neurol..

[60] Leentjens AF (2011). Symptomatology and markers of anxiety disorders in Parkinson’s disease: a cross-sectional study. Mov. Disord..

[61] Mondolo F (2007). Evaluation of anxiety in Parkinson’s disease with some commonly used rating scales. Neurol. Sci..

[62] Taschereau-Dumouchel V, Kawato M, Lau H (2020). Multivoxel pattern analysis reveals dissociations between subjective fear and its physiological correlates. Mol. Psychiatry.

[63] Upneja A, Paul BS, Jain D, Choudhary R, Paul G (2021). Anxiety in Parkinson’s disease: correlation with depression and quality of life. J. Neurosci. Rural Pract..

[64] Rutten S (2015). Anxiety in Parkinson’s disease: symptom dimensions and overlap with depression and autonomic failure. Parkinsonism Relat. Disord..

[65] Beck AT, Epstein N, Brown G, Steer RA (1988). An inventory for measuring clinical anxiety: psychometric properties. J. Consult. Clin. Psychol..

[66] Leentjens AF (2014). The Parkinson Anxiety Scale (PAS): development and validation of a new anxiety scale. Mov. Disord..

[67] Moriyama TS (2016). Diagnosing social anxiety in Parkinson’s disease: characteristics and frequencies according to two diagnostic criteria. Arch. Clin. Psychiatry (São Paulo).

[68] Segers K, Benoit F, Meyts JM, Surquin M (2020). Anxiety symptoms are quantitatively and qualitatively different in dementia with Lewy bodies than in Alzheimer’s disease in the years preceding clinical diagnosis. Psychogeriatrics.

[69] Breitve MH (2016). A longitudinal study of anxiety and cognitive decline in dementia with Lewy bodies and Alzheimer’s disease. Alzheimer’s Res. Ther..

[70] Borroni B, Agosti C, Padovani A (2008). Behavioral and psychological symptoms in dementia with Lewy-bodies (DLB): frequency and relationship with disease severity and motor impairment. Arch. Gerontol. Geriatr..

[71] Zhang LY (2018). Depression and anxiety in multiple system atrophy. Acta Neurol. Scand..

[72] Schrag A (2010). A comparison of depression, anxiety, and health status in patients with progressive supranuclear palsy and multiple system atrophy. Mov. Disord..

[73] Jecmenica-Lukic M (2021). The profile and evolution of neuropsychiatric symptoms in multiple system atrophy: self- and caregiver report. J. Neuropsychiatry Clin. Neurosci..

[74] Kao AW (2009). Cognitive and neuropsychiatric profile of the synucleinopathies: Parkinson disease, dementia with Lewy bodies, and multiple system atrophy. Alzheimer Dis. Assoc. Disord..

[75] Forbes EJ (2021). Defining atypical anxiety in Parkinson’s disease. Mov. Disord. Clin. Pract..

[76] Seritan AL, Rienas C, Duong T, Delucchi K, Ostrem JL (2019). Ages at onset of anxiety and depressive disorders in Parkinson’s disease. J. Neuropsychiatry Clin. Neurosci..

[77] Shiba M (2000). Anxiety disorders and depressive disorders preceding Parkinson’s disease: a case-control study. Mov. Disord..

[78] Dissanayaka NN (2010). Anxiety disorders in Parkinson’s disease: prevalence and risk factors. Mov. Disord..

[79] Lin CH, Lin JW, Liu YC, Chang CH, Wu RM (2015). Risk of Parkinson’s disease following anxiety disorders: a nationwide population-based cohort study. Eur. J. Neurol..

[80] Bower JH (2010). Anxious personality predicts an increased risk of Parkinson’s disease. Mov. Disord..

[81] Rana AQ, Ansari H, AR MQ, Rahman E (2018). Impact of progression of Parkinson’s disease and various other factors on generalized anxiety disorder. J. Neurosci. Rural Pract..

[82] Toloraia K (2022). Anxiety, depression, and apathy as predictors of cognitive decline in patients with Parkinson’s disease—a Three-Year Follow-Up Study. Front. Neurol..

[83] Horne KL (2021). Neuropsychiatric symptoms are associated with dementia in Parkinson’s disease but not predictive of it. Mov. Disord. Clin. Pract..

[84] Dissanayaka NN (2022). Anxiety disorders are associated with verbal memory impairment in patients with Parkinson’s disease without dementia. J. Neurol..

[85] van der Velden RMJ, Broen MPG, Kuijf ML, Leentjens AFG (2018). Frequency of mood and anxiety fluctuations in Parkinson’s disease patients with motor fluctuations: a systematic review. Mov. Disord..

[86] Carey G (2020). Anxiety in Parkinson’s disease is associated with changes in the brain fear circuit. Parkinsonism Relat. Disord..

[87] Carey G (2021). Neuroimaging of anxiety in Parkinson’s disease: a systematic review. Mov. Disord..

[88] Dan R (2017). Separate neural representations of depression, anxiety and apathy in Parkinson’s disease. Sci. Rep..

[89] Tinaz S (2021). Distinct neural circuits are associated with subclinical neuropsychiatric symptoms in Parkinson’s disease. J. Neurol. Sci..

[90] Oosterwijk CS, Vriend C, Berendse HW, van der Werf YD, van den Heuvel OA (2018). Anxiety in Parkinson’s disease is associated with reduced structural covariance of the striatum. J. Affect. Disord..

[91] Zhang P (2023). Altered fractional amplitude of low-frequency fluctuation in anxious Parkinson’s disease. Brain Sci..

[92] De Micco R (2021). Connectivity correlates of anxiety symptoms in drug-naive Parkinson’s disease patients. Mov. Disord..

[93] Wang Y (2021). Altered cerebellum functional network on newly diagnosed drug-naive Parkinson’s disease patients with anxiety. Transl. Neurosci..

[94] Criaud M (2021). Anxiety in Parkinson’s disease: abnormal resting activity and connectivity. Brain Res..

[95] Betrouni N (2022). Anxiety in Parkinson’s disease: a resting-state high density EEG study. Neurophysiol. Clin..

[96] Flores-Cuadrado A, Ubeda-Banon I, Saiz-Sanchez D, Martinez-Marcos A (2017). alpha-Synucleinopathy in the human amygdala in Parkinson disease: differential vulnerability of somatostatin- and parvalbumin-expressing neurons. J. Neuropathol. Exp. Neurol..

[97] Floresco SB, Tse MT (2007). Dopaminergic regulation of inhibitory and excitatory transmission in the basolateral amygdala-prefrontal cortical pathway. J. Neurosci..

[98] Torres ERS (2021). Alpha-synuclein pathology, microgliosis, and parvalbumin neuron loss in the amygdala associated with enhanced fear in the Thy1-aSyn model of Parkinson’s disease. Neurobiol. Dis..

[99] Harding AJ, Stimson E, Henderson JM, Halliday GM (2002). Clinical correlates of selective pathology in the amygdala of patients with Parkinson’s disease. Brain.

[100] Milham MP (2005). Selective reduction in amygdala volume in pediatric anxiety disorders: a voxel-based morphometry investigation. Biol. Psychiatry.

[101] Hayano F (2009). Smaller amygdala is associated with anxiety in patients with panic disorder. Psychiatry Clin. Neurosci..

[102] Flores-Cuadrado A, Ubeda-Banon I, Saiz-Sanchez D, de la Rosa-Prieto C, Martinez-Marcos A (2015). alpha-Synuclein staging in the amygdala of a Parkinson’s disease model: cell types involved. Eur. J. Neurosci..

[103] Bourgouin PA (2019). Gray matter substrates of depressive and anxiety symptoms in idiopathic REM sleep behavior disorder. Parkinsonism Relat. Disord..

[104] Erro R (2012). Anxiety is associated with striatal dopamine transporter availability in newly diagnosed untreated Parkinson’s disease patients. Parkinsonism Relat. Disord..

[105] Weintraub D (2005). Striatal dopamine transporter imaging correlates with anxiety and depression symptoms in Parkinson’s disease. J. Nucl. Med..

[106] de Oliveira AR (2011). Conditioned fear is modulated by D2 receptor pathway connecting the ventral tegmental area and basolateral amygdala. Neurobiol. Learn. Mem..

[107] Tang W, Kochubey O, Kintscher M, Schneggenburger R (2020). A VTA to basal amygdala dopamine projection contributes to signal salient somatosensory events during fear learning. J. Neurosci..

[108] Brandão ML, Coimbra NC (2019). Understanding the role of dopamine in conditioned and unconditioned fear. Rev. Neurosci..

[109] Maillet A (2016). The prominent role of serotonergic degeneration in apathy, anxiety and depression in de novo Parkinson’s disease. Brain.

[110] Ballanger B (2012). Role of serotonergic 1A receptor dysfunction in depression associated with Parkinson’s disease. Mov. Disord..

[111] Reisine TD, Fields JZ, Yamamura HI (1977). Neurotransmitter receptor alterations in Parkinson’s disease. Life Sci..

[112] Bocchio M, McHugh SB, Bannerman DM, Sharp T, Capogna M (2016). Serotonin, amygdala and fear: assembling the puzzle. Front. Neural Circuits.

[113] Menza MA, Palermo B, DiPaola R, Sage JI, Ricketts MH (1999). Depression and anxiety in Parkinson’s disease: possible effect of genetic variation in the serotonin transporter. J. Geriatr. Psychiatry Neurol..

[114] Joling M, van den Heuvel OA, Berendse HW, Booij J, Vriend C (2018). Serotonin transporter binding and anxiety symptoms in Parkinson’s disease. J. Neurol. Neurosurg. Psychiatry.

[115] Chen JJ, Marsh L (2014). Anxiety in Parkinson’s disease: identification and management. Ther. Adv. Neurol. Disord..

[116] Vieira JCF (2019). Anxiety-like behavior induced by 6-OHDA animal model of Parkinson’s disease may be related to a dysregulation of neurotransmitter systems in brain areas related to anxiety. Behav. Brain Res..

[117] Du CX (2018). Involvement of prelimbic 5-HT(7) receptors in the regulation of anxiety-like behaviors in hemiparkinsonian rats. Neurol. Res..

[118] Liu KC (2019). Activation and blockade of dorsal hippocampal Serotonin(6) receptors regulate anxiety-like behaviors in a unilateral 6-hydroxydopamine rat model of Parkinson’s disease. Neurol. Res..

[119] Chiavegatto S (2009). Expression of alpha-synuclein is increased in the hippocampus of rats with high levels of innate anxiety. Mol. Psychiatry.

[120] Chesselet M-F, Richter F (2011). Modelling of Parkinson’s disease in mice. Lancet Neurol..

[121] McDowell K, Chesselet MF (2012). Animal models of the non-motor features of Parkinson’s disease. Neurobiol. Dis..

[122] Dujardin K, Sgambato V (2020). Neuropsychiatric disorders in Parkinson’s disease: what do we know about the role of dopaminergic and non-dopaminergic systems?. Front. Neurosci..

[123] Carvalho MM (2013). Behavioral characterization of the 6-hydroxidopamine model of Parkinson’s disease and pharmacological rescuing of non-motor deficits. Mol. Neurodegener..

[124] Antunes MS (2020). Hesperidin ameliorates anxiety-depressive-like behavior in 6-OHDA model of Parkinson’s disease by regulating striatal cytokine and neurotrophic factors levels and dopaminergic innervation loss in the striatum of mice. Mol. Neurobiol..

[125] Avila G, Picazo O, E C, M, M GR (2020). Reduction of dopaminergic transmission in the globus pallidus increases anxiety-like behavior without altering motor activity. Behav. Brain Res..

[126] Drui G (2014). Loss of dopaminergic nigrostriatal neurons accounts for the motivational and affective deficits in Parkinson’s disease. Mol. Psychiatry.

[127] Ferrazzo S (2019). Increased anxiety-like behavior following circuit-specific catecholamine denervation in mice. Neurobiol. Dis..

[128] Zhang J (2022). Activation of AMPA receptors in the lateral habenula produces anxiolytic effects in a rat model of Parkinson’s disease. Front. Pharm..

[129] Masini, D. et al. A guide to the generation of a 6-hydroxydopamine mouse model of Parkinson’s disease for the study of non-motor symptoms. *Biomedicines* **9**, 10.3390/biomedicines9060598 (2021). 10.3390/biomedicines9060598 PMC8227396 34070345

[130] Bove J, Prou D, Perier C, Przedborski S (2005). Toxin-induced models of Parkinson’s disease. NeuroRx.

[131] Shin KS (2014). Effects of gypenosides on anxiety disorders in MPTP-lesioned mouse model of Parkinson׳s disease. Brain Res..

[132] Yan J (2020). Atorvastatin improves motor function, anxiety and depression by NOX2-mediated autophagy and oxidative stress in MPTP-lesioned mice. Aging (Albany, NY).

[133] Ujvári B (2022). Neurodegeneration in the centrally-projecting Edinger–Westphal nucleus contributes to the non-motor symptoms of Parkinson’s disease in the rat. J. Neuroinflamm..

[134] Tsarouchi M, Fanarioti E, Karathanos VT, Dermon CR (2023). Protective effects of currants (*Vitis vinifera*) on corticolimbic serotoninergic alterations and anxiety-like comorbidity in a rat model of Parkinson’s disease. Int. J. Mol. Sci..

[135] Tinakoua A (2015). The impact of combined administration of paraquat and maneb on motor and non-motor functions in the rat. Neuroscience.

[136] Czerniczyniec A, Karadayian AG, Bustamante J, Cutrera RA, Lores-Arnaiz S (2011). Paraquat induces behavioral changes and cortical and striatal mitochondrial dysfunction. Free Radic. Biol. Med..

[137] Yan J (2020). Simvastatin improves behavioral disorders and hippocampal inflammatory reaction by NMDA-mediated anti-inflammatory function in MPTP-treated mice. Cell. Mol. Neurobiol..

[138] Yun SP (2018). Block of A1 astrocyte conversion by microglia is neuroprotective in models of Parkinson’s disease. Nat. Med..

[139] Johnson ME (2021). Heterozygous GBA D409V and ATP13a2 mutations do not exacerbate pathological alpha-synuclein spread in the prodromal preformed fibrils model in young mice. Neurobiol. Dis..

[140] Karampetsou M (2017). Phosphorylated exogenous alpha-synuclein fibrils exacerbate pathology and induce neuronal dysfunction in mice. Sci. Rep..

[141] Luk KC (2012). Pathological alpha-synuclein transmission initiates Parkinson-like neurodegeneration in nontransgenic mice. Science.

[142] Stoyka LE (2020). Behavioral defects associated with amygdala and cortical dysfunction in mice with seeded alpha-synuclein inclusions. Neurobiol. Dis..

[143] Burtscher J (2019). Chronic corticosterone aggravates behavioral and neuronal symptomatology in a mouse model of alpha-synuclein pathology. Neurobiol. Aging.

[144] Boutros SW, Raber J, Unni VK (2021). Effects of alpha-synuclein targeted antisense oligonucleotides on lewy body-like pathology and behavioral disturbances induced by injections of pre-formed fibrils in the mouse motor cortex. J. Parkinson’s Dis..

[145] Graham DR, Sidhu A (2010). Mice expressing the A53T mutant form of human alpha-synuclein exhibit hyperactivity and reduced anxiety-like behavior. J. Neurosci. Res..

[146] Rothman SM (2013). Neuronal expression of familial Parkinson’s disease A53T α-synuclein causes early motor impairment, reduced anxiety and potential sleep disturbances in mice. J. Parkinson’s Dis..

[147] Freichel C (2007). Age-dependent cognitive decline and amygdala pathology in alpha-synuclein transgenic mice. Neurobiol. Aging.

[148] Yamakado H (2012). alpha-Synuclein BAC transgenic mice as a model for Parkinson’s disease manifested decreased anxiety-like behavior and hyperlocomotion. Neurosci. Res..

[149] Kohl Z (2016). Severely impaired hippocampal neurogenesis associates with an early serotonergic deficit in a BAC α-synuclein transgenic rat model of Parkinson’s disease. Neurobiol. Dis..

[150] Uemura N (2021). α-Synuclein spread from olfactory bulb causes hyposmia, anxiety, and memory loss in BAC-SNCA mice. Mov. Disord..

[151] Chesselet MF (2012). A progressive mouse model of Parkinson’s disease: the Thy1-aSyn (“Line 61”) mice. Neurotherapeutics.

[152] Richter F, Stanojlovic M, Kaufer C, Gericke B, Feja M (2023). A mouse model to test novel therapeutics for Parkinson’s disease: an update on the Thy1-aSyn (“line 61”) mice. Neurotherapeutics.

[153] George S (2008). Alpha-synuclein transgenic mice exhibit reduced anxiety-like behaviour. Exp. Neurol..

[154] Peña-Oliver Y, Buchman VL, Stephens DN (2010). Lack of involvement of alpha-synuclein in unconditioned anxiety in mice. Behav. Brain Res.

[155] Deusser J (2015). Serotonergic dysfunction in the A53T alpha-synuclein mouse model of Parkinson’s disease. J. Neurochem..

[156] Levigoureux E, Bouillot C, Baron T, Zimmer L, Lancelot S (2019). PET imaging of the influence of physiological and pathological alpha-synuclein on dopaminergic and serotonergic neurotransmission in mouse models. CNS Neurosci. Ther..

[157] Butkovich LM (2020). Transgenic mice expressing human α-synuclein in noradrenergic neurons develop locus ceruleus pathology and nonmotor features of Parkinson’s disease. J. Neurosci..

[158] Stanojlovic M, Pallais JP, Kotz CM (2019). Chemogenetic modulation of orexin neurons reverses changes in anxiety and locomotor activity in the A53T mouse model of Parkinson’s disease. Front. Neurosci..

[159] Jiao L (2020). Tau knockout exacerbates degeneration of parvalbumin-positive neurons in substantia nigra pars reticulata in Parkinson’s disease-related alpha-synuclein A53T mice. FASEB J..

[160] Felger JC (2018). Imaging the role of inflammation in mood and anxiety-related disorders. Curr. Neuropharmacol..

[161] Li H, Wang H, Zhang L, Wang M, Li Y (2021). Dl-3-n-butylphthalide alleviates behavioral and cognitive symptoms via modulating mitochondrial dynamics in the A53T-α-synuclein mouse model of Parkinson’s disease. Front. Neurosci..

[162] Farrell KF (2013). Non‐motor parkinsonian pathology in aging A53T α‐Synuclein mice is associated with progressive synucleinopathy and altered enzymatic function. J. Neurochem..

[163] Wang W (2018). Genomic DNA levels of mutant alpha-synuclein correlate with non-motor symptoms in an A53T Parkinson’s disease mouse model. Neurochem. Int..

[164] Visanji NP (2016). α-Synuclein-based animal models of Parkinson’s disease: challenges and opportunities in a new era. Trends Neurosci..

[165] Gómez-Benito M (2020). Modeling Parkinson’s disease with the alpha-synuclein protein. Front. Pharm..

[166] Bichler Z, Lim HC, Zeng L, Tan EK (2013). Non-motor and motor features in LRRK2 transgenic mice. PLoS ONE.

[167] Rial D (2014). Behavioral phenotyping of Parkin-deficient mice: looking for early preclinical features of Parkinson’s disease. PLoS ONE.

[168] Li M (2019). Impaired D2 receptor-dependent dopaminergic transmission in prefrontal cortex of awake mouse model of Parkinson’s disease. Brain.

[169] Duan K (2021). Mitophagy in the basolateral amygdala mediates increased anxiety induced by aversive social experience. Neuron.

[170] Hoffmeister JD, Kelm-Nelson CA, Ciucci MR (2021). Quantification of brainstem norepinephrine relative to vocal impairment and anxiety in the Pink1−/− rat model of Parkinson disease. Behav. Brain Res..

[171] Hoffmeister JD, Kelm-Nelson CA, Ciucci MR (2022). Manipulation of vocal communication and anxiety through pharmacologic modulation of norepinephrine in the Pink1−/− rat model of Parkinson disease. Behav. Brain Res.

[172] Gaztanaga W (2021). Do benzodiazepines impair motor and nonmotor symptoms in a sample of Parkinson’s disease patients?. Cureus.

[173] Ferreira RM (2018). The effect of resistance training on the anxiety symptoms and quality of life in elderly people with Parkinson’s disease: a randomized controlled trial. Arq. Neuropsiquiatr..

[174] Kwok JYY (2019). Effects of mindfulness yoga vs stretching and resistance training exercises on anxiety and depression for people with Parkinson disease: a Randomized Clinical Trial. JAMA Neurol..

[175] Zhang T (2017). MPTP-induced dopamine depletion in basolateral amygdala via decrease of D2R activation suppresses GABA(A) receptors expression and LTD induction leading to anxiety-like behaviors. Front. Mol. Neurosci..

[176] Tran L, Lasher BK, Young KA, Keele NB (2013). Depletion of serotonin in the basolateral amygdala elevates glutamate receptors and facilitates fear-potentiated startle. Transl. Psychiatry.

[177] Liu WZ (2020). Identification of a prefrontal cortex-to-amygdala pathway for chronic stress-induced anxiety. Nat. Commun..

[178] Yasmin F (2020). Stress-induced modulation of endocannabinoid signaling leads to delayed strengthening of synaptic connectivity in the amygdala. PNAS.

[179] Elefante, C. et al. Prevalence and clinical correlates of comorbid anxiety and panic disorders in patients with Parkinson’s disease. *J. Clin. Med.* **10**, 10.3390/jcm10112302 (2021). 10.3390/jcm10112302 PMC8198165 34070549

[180] Benli E (2021). Effect of bladder dysfunction on development of depression and anxiety in Parkinson’s disease. Arch. Ital. Urol. Androl..

[181] Zhao C, Cai H, Wang H, Ge Z (2021). Correlation between serum renin-angiotensin system (RAS) level and depression and anxiety symptoms in patients with Parkinson’s disease. Saudi J. Biol. Sci..

[182] Rutten S, van Wegen EEH, Ghielen I, Schoon B, van den Heuvel OA (2021). Symptom dimensions of anxiety in Parkinson’s disease: replication study in a neuropsychiatric patient population. Clin. Parkinsonism Relat. Disord..

[183] Wang J (2023). Common and distinct roles of amygdala subregional functional connectivity in non-motor symptoms of Parkinson’s disease. npj Parkinson’s Dis..

[184] Weintraub D (2015). Cognitive performance and neuropsychiatric symptoms in early, untreated Parkinson’s disease. Mov. Disord..

[185] Paumier KL (2013). Behavioral characterization of A53T mice reveals early and late stage deficits related to Parkinson’s disease. PLoS ONE.

[186] Kyser TL (2019). Characterization of motor and non-motor behavioral alterations in the Dj-1 (PARK7) knockout rat. J. Mol. Neurosci..

